# Blood-brain barrier permeability following traumatic brain injury

**DOI:** 10.1186/cc10919

**Published:** 2012-03-20

**Authors:** M Jungner, P Bentzer

**Affiliations:** 1 Lund University, Lund, Sweden

## Introduction

Brain edema and intracranial hypertension is deleterious after traumatic brain injury (TBI), but the underlying pathophysiology is complex and poorly understood. One major subject of controversy is the time course and extent of blood-brain barrier dysfunction following trauma, and previous studies in humans have only provided semi-quantitative data. The objective of the present study was therefore to quantify changes in blood-brain barrier permeability in the early course of TBI.

## Methods

Seventeen nonconsecutive brain trauma patients and two controls were included in this prospective observational study. Following i.v. injection of iohexol and CT perfusion scans, patients were scanned eight times from 4 to 25 minutes. The blood-to-brain transfer constant (K_i_) for iohexol, reflecting permeability and area available for diffusion, was calculated by Patlak plot analysis of the enhancement curves of intracerebral large venous vessels and pericontusional brain parenchyma.

## Results

**Figure 1:**
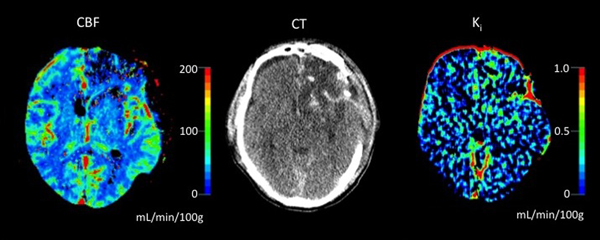
**CBF (left) and permeability (right) maps, and contrast-enhanced CT scan (middle)**.

Fourteen patients were included within 1 day and three were included within 5 days of the injury. In nonischemic tissue surrounding contusions and hematomas, K_i _was focally increased in 11 of all included trauma patients and in six of seven patients with raised intracranial pressure. In noninjured areas and in controls, K_i _was about 0.06 ml/minute/100 g and increased by 100 to 2,000% in pericontusional tissue. See Figure 1.

## Conclusion

TBI is associated with early focal increases in blood-brain barrier permeability. The results suggest that in the injured brain, capillary hydrostatic and oncotic pressures are likely to influence edema formation.
